# Feeding practices of low birth weight Brazilian infants and associated factors

**DOI:** 10.11606/s1518-8787.2020054001028

**Published:** 2020-01-23

**Authors:** Naiá Ortelan, Daniela Almeida Neri, Maria Helena D’Aquino Benicio

**Affiliations:** I Universidade de São Paulo Faculdade de Saúde Pública Departamento de Nutrição em Saúde Pública São PauloSP Brasil Universidade de São Paulo. Faculdade de Saúde Pública. Departamento de Nutrição em Saúde Pública. São Paulo, SP, Brasil; II Universidade de São Paulo Faculdade de Saúde Pública São PauloSP Brasil Universidade de São Paulo. Faculdade de Saúde Pública. Núcleo de Pesquisas Epidemiológicas em Nutrição e Saúde (NUPENS). São Paulo, SP, Brasil

**Keywords:** Infant, Low Birth Weight, Infant Food, Infant Nutrition, Diet, Food, and Nutrition, Ultraprocessed Food

## Abstract

**OBJECTIVE:**

To characterize complementary feeding and to analyze the influence of individual and contextual factors on dietary practices of low birth weight infants.

**METHODS:**

This cross-sectional study included 2,370 low birth weight infants aged 6 to 12 months included in the Breastfeeding Prevalence Survey in Brazilian Municipalities (2008), which covered the 26 state capitals, the Federal District and 37 municipalities. Dietary practices were assessed using two indicators: I) dietary diversity, characterized by the consumption of five food groups: meat, beans, vegetables, fruit and milk; II) consumption of ultra-processed foods, characterized by the ingestion of at least one of the following foods on the day prior to the survey: soda, or processed juice, or cookie, cracker and crisps. The covariates of interest were the socioeconomic characteristics of infants, mothers and health services. The contextual factor was the “municipal prevalence of child undernutrition.” The individualized effect of the study factors on outcomes was assessed by multilevel Poisson regression.

**RESULTS:**

Approximately 59% of infants consumed ultra-processed foods, while 29% had diverse feeding. Mothers living in municipalities with child undernutrition prevalence below 10%, with higher education and working outside the home were more likely to offer dietary diversity. Consumption of ultra-processed foods was higher among infants living in municipalities with child undernutrition prevalence below 10%, whose mothers were younger and multiparous.

**CONCLUSIONS:**

The low prevalence of diverse feeding combined with the high prevalence of ultra-processed food consumption characterizes the low quality of feeding of low birth weight Brazilian infants. Individual and contextual factors impact the feeding quality of this population, suggesting the need for effective strategies to increase the consumption of fresh and minimally processed foods and decrease the consumption of ultra-processed foods by this vulnerable population.

## INTRODUCTION

Low birth weight (LBW, < 2,500 g) is considered a major global public health problem because it is associated with high morbidity and mortality, risk of poor growth and specific deficiencies, as well as rapid weight gain, cognitive problems and behavioral changes throughout life^1^. Despite major advances in prenatal care in recent years, its prevalence has remained close to 8% since 2000 in Brazil, and the worldwide incidence has remained at 15% between 2008 and 2012^a^.

LBW occurs as a consequence of prematurity (gestational age less than 37 weeks), intrauterine growth restriction (IUGR) or the combination of both, being mainly related to conditions of poverty, undernutrition and insufficient diet^2 , 3^. When combined with IUGR, LBW makes children very vulnerable to nutritional deficiencies, of both shortage and excess. Epidemiological and clinical studies in children small for gestational age have found a strong association between disorders occurring in the fetal life (nutrient scarcity) or in the early stages of extrauterine life (rapid nutritional recovery early in the postnatal life) and the onset of chronic noncommunicable diseases (CNCD) throughout life, such as obesity, hypertension, cardiovascular disease, insulin resistance, and type 2 diabetes^4 - 6^.

Although there is no consensus on the nutritional needs of LBW infants, it is known that once they reach full feeding and have adequate weight gain for hospital discharge, nutritional recommendations and health monitoring become similar to those for the general population. Thus, it is assumed that, during the first two years of life, children with LBW are exposed to the same feeding and nutrition situation of children under two years of the general Brazilian population, in a scenario marked by: early introduction of complementary feeding (CF)^7^, low consumption of appropriate meals in consistency and texture for age, and high consumption of ultra-processed foods (UPF)^7^. These foods are ready-to-eat industrial formulations made entirely or mostly of substances extracted from foods (oils, fats, sugar, proteins), derived from food constituents (hydrogenated fats, modified starch) or synthesized in the laboratory based on organic materials (colorants, flavorings, flavor enhancers and other additives used to alter sensory properties)^8^, for example, soft drinks and other sweetened beverages, stuffed cookies, crisps, instant noodles, ice cream, treats, sugary breakfast cereals, cereal bars, sausages, hamburgers (not handmade) and loaves, hamburger and hot dog buns, among others.

Promoting adequate and healthy complementary feeding has numerous benefits because: it is considered the third most effective action with the potential to prevent 6% of all under-five deaths worldwide^9^; is associated with the establishment of healthy eating habits, which are reflected in childhood^10 , 11^and adulthood^12^; is among the modifiable risk factors for obesity in children^13^; helps children reach their developmental potential and become healthy adults with greater intellectual and productive capacity^14^; favors sustainable economic development and poverty reduction of a nation^15^.

Thus, it is essential to monitor whether the feeding of LBW children meets their nutritional needs and reduces the risk of chronic disease. From this perspective, the first survey called Breastfeeding Prevalence Survey (BPS) in the Brazilian Capitals and Federal District^16^wasconducted in 1999 during the national vaccination campaign and, in 2008, BPS II^7^, justified by the need for an analysis of the evolution of eating practices of children under 1 year, in view of the various actions developed within the national policy. However, to date there is no knowledge about these eating practices in the population of low birth weight infants. These data are also scarce in the international literature.

In order to differentiate this study from the others, we defined a study population rather than a variable, highlighting the originality centered on the emphasis on LBW rather than conducting yet another “LBW versus non-LBW” prevalence study. Thus, it was possible to explore the profile and differentials of this vulnerable population, emphasizing its importance. Given the double burden of nutritional risks to which LBW Brazilian children are exposed, the heterogeneity of income and education in Brazil, the need for monitoring this population, and the availability of BPS II data, the present study had as its main objective to characterize the CF of low birth weight Brazilian infants aged from six months and 0 days to 11 months and 29 days (i.e. from complete six months to incomplete twelve months) and to analyze the influence of individual and contextual factors on eating practices related to dietary diversity and consumption of ultra-processed foods. The results may be useful and contribute significantly to the improvement of public policies aimed at improving child feeding in this group.

## METHODS

This cross-sectional study is part of the BPS in Brazilian Municipalities (2008) conducted in 26 Brazilian state capitals, the Federal District, and 37 other municipalities with a population of children under one year of age exceeding 4 thousand, to assess the breastfeeding and CF situation in the country. Data were obtained during the 2008 National Vaccination Campaign using a closed questionnaire that included questions on the sociodemographic characteristics of mothers or guardians and children, as well as on the consumption of breast milk, other types of milk and other foods on the day before the survey. Details on sample size calculation and data collection can be found elsewhere^7 , 17^.

**Figure 1:**
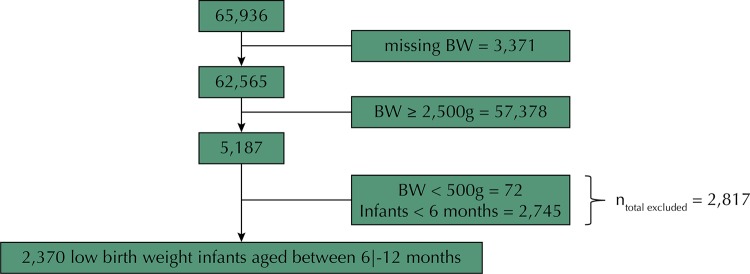
Flowchart representing the population definitions of this study. BW: birth weight

The present study included 2,370 infants born underweight and aged six months and 0 days to 11 months and 29 days (due to the recommendation of exclusive breastfeeding up to six months) living in 64 Brazilian municipalities. The population definition of this article can be found in the appendix ( Figure 1 ).

Because it is research conducted with complex probabilistic sampling, specific procedures were used for its analysis. Due to population differences across the municipalities studied, each level corresponded to a different sample fraction, represented by the estimated sample size on the number of children to be vaccinated. The inverse of this fraction was applied as children’s weight in each municipality^7^.

We evaluated two dimensions of interest in the feeding of vulnerable populations: i) the fraction composed of non- or minimally processed foods, which is positively related to nutritional adequacy (positive dimension); ii) the fraction composed by UPF, which is positively related to high energy density, excess free sugar and saturated and trans fat, and protein and micronutrient inadequacy (negative dimension).

To assess dietary diversity we used an indicator adapted to that proposed by the World Health Organization (WHO)^18^. In the present study, diversity was characterized as the consumption of five food groups in the last 24 hours: meat, beans, vegetables, fruits and milk (breast or other), as proposed in other studies^19 , 20^.

UPF consumption assessment was based on the NOVA classification, which categorizes foods according to the extent and purpose of their processing^21^, and on the set of foods considered ultra-processed in the BPS questionnaire: soda, processed juice and crackers, cookies and crisps.

Thus, the outcomes of the present study were: i) “dietary diversity,” defined as the consumption of five food groups at least once on the day prior to the survey, and ii) “consumption of UPF,” when the infant consumed at least one of the foods mentioned above on the day prior to the survey. Both dependent variables were dichotomized into 0 (no) and 1 (yes).

The covariates of interest corresponded to the socioeconomic characteristics (represented by the proxy maternal education in years of schooling: ≤ 8, 9|–12, ≥ 12), to the infants characteristics (age: 6|–7 months, 7|–8 months, 8|–9 months, 9|–10 months, 10|–11 months, 11|–12 months; sex: male, female), to the mothers characteristics (age range: < 20, 20|–35, ≥ 35; working outside: no, yes, parity: primiparous, multiparous) and to the health services (outpatient follow-up: private or insurance plan, public network; type of delivery: vaginal, cesarean section). The first contextual factor studied regarding the municipality was the municipal prevalence of child undernutrition (≥ 10%, < 10%), a variable used as a proxy for poverty and estimated by Benicio et al.^22^from data from the 2006 National Demographic and Health Survey (NDHS) and the 2000 Demographic Census sample. Child undernutrition was measured by the height deficit for age below -2 Z-scores of the 2006 WHO growth pattern. Estimates of child undernutrition prevalence were produced for each of the 5,507 Brazilian municipalities in 2000. The method used to obtain these estimates was based on the development of individual statistical prediction models using multilevel analysis based on the 2006 NDHS, with inclusion of predictive variables measured similarly in both surveys. Prevalence was then estimated by the average individual probability of children living in each municipality studied by the 2000 Census sample. The second contextual factor was the municipality’s human development index (HDI), used as a direct indicator to refer to the socioeconomic status of the 64 municipalities studied in 2010^b^, stratified in ascending order of classification: low (0.500–0.599) + medium (0.600–0.699), high (0.700–0.799), and very high (> 0.800).

**Figure 2:**
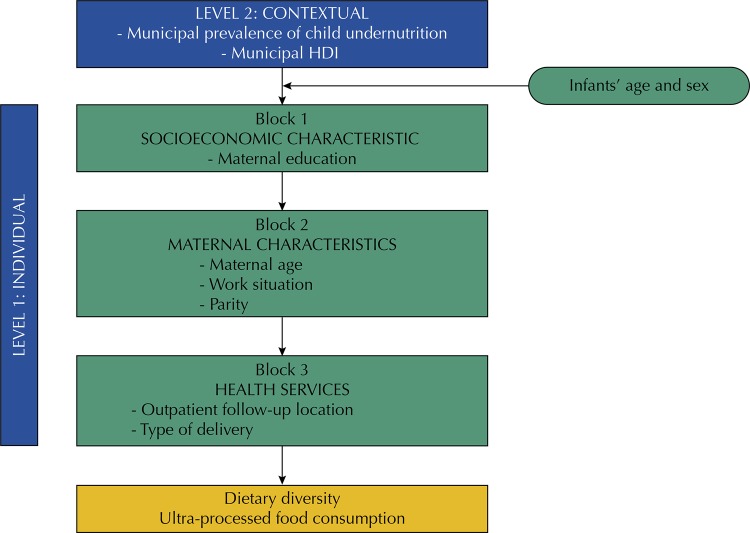
Conceptual framework for investigating individual and contextual factors of dietary diversity and ultra-processed foods consumption. HDI: human development index

The conceptual model used is depicted in Figure 2 . It shows that the hierarchical modeling strategy was used to include the individual variables in the model. In this strategy, the hierarchy of the independent variables is established in a conceptual framework, and the choice of criteria to select them requires knowledge about temporal precedence and biological and social determinants, rather than considering only the statistical aspect. Hierarchization was maintained throughout the analysis, allowing the selection of the variables most strongly associated with the outcomes of interest^23^.

The association between the independent variables and the outcomes was initially evaluated using Poisson regression adjusted only for infant age. Thus, we selected those that could influence the outcomes. The effect of these, free of confusion generated by the others, was evaluated by multilevel Poisson regression, used according to the hierarchical organization of the population in relation to socioeconomic, infant, maternal and health service (Level 1) characteristics, considering the context (Level 2) and the existence of intragroup correlation.

The final model retained as adjustment variables only those with p <0.15 in the analysis adjusted for infant age. The selection of the variables that made up the final model followed the backward selection process: in the first stage of the modeling, the contextual variables adjusted for infant age and sex for both outcomes (Level 2) were included; in the following three stages, those related to socioeconomic (Block 1) or maternal (Block 2) and health services (Block 3) characteristics, all of them Level 1. Covariates with more than two categories were introduced into the model as ordinal qualitative variables to estimate the linear trend p-value. Prevalence ratio (PR) values and their respective confidence intervals (95%CI) were presented. The association between study factor and outcome was considered significant when p < 0.05. Evaluation of the fit quality of the multilevel model was verified by the -2loglikelihood test. All analyses were performed using Stata/SE 14.1 software.

This project was approved by the Research Ethics Committee of the Faculdade de Saúde Pública of Universidade de São Paulo, under opinion number 58147216.1.0000.5421.

## RESULTS

Table 1 shows the sample and feeding characteristics of the infants studied. The median infant age was 8.9 months and birth weight 2,230 g, with 72% born weighing between 2,000 and 2,499 g. About 67% of the mothers were between 20 and 35 years old, 71% were not working outside the home and 54% were primiparous. The public network was the main outpatient follow-up (61.9%) and cesarean section was the most prevalent type of delivery (55.3%). Approximately 85% of infants lived in municipalities with child undernutrition prevalence below 10%, and 53% in municipalities with a high HDI. The prevalence of breastfeeding on the day before the survey was 54.5%. Regarding fluid consumption on the day before the survey, the highest prevalence found was for water (89.2%), followed by breast milk, non-breast milk or porridge (80.9%), some other type of non-breast milk (71.5%), and natural juice (70.8%). Thus, the probability of the infant receiving breast milk was lower than that found for other milks and porridge. Regarding the consumption of semi-solid foods on the day before the survey, there was a higher prevalence of vegetables (93.5%), followed by meat (77.7%), fruit (74.1%) and beans (67.3%). Regarding dietary practices, despite the high consumption of the main meal at lunch and/or dinner (84.5%), there was high consumption of UPF (58.9%), and only 28.5% of the study population received the five food groups at least once in the last 24 hours.

**Table 1:** Sample and feeding characteristics of low birth weight infants aged 6 to 11 months and 29 days. Breastfeeding Prevalence Survey in Brazilian Municipalities, 2008 (n = 2,370).

Caracteristics of sample	N_total _or median^a^	% by category or interquartile interval^b^
LEVEL 1. Infants, maternal, socioeconomic and health service characteristic		

Block 1 – Socioeconomic characteristic		

Maternal education (years)		
≤ 8	831	40.4
9–12	890	47.1
≥ 12	248	12.5

Block 2 – Infants and maternal characteristics		

Sex		
Male	1,079	43.7
Female	1,291	56.3
Age of infants (months)	8,9^a^	7.3–10.5^b^
Birth weight (g)	2,230^a^	1.960–2.380^b^
< 1,500	250	11.8
1,500–1,999	380	16.2
2,000–2,499	1,740	72.0
Maternal age range (years)		
< 20	384	18.1
20–35	1,339	66.9
≥ 35	259	15.0
Maternal work		
Does not work outside home	1,381	70.7
Works outside home	514	29.4
Parity		
Primiparous	1,058	54.4
Multiparous	886	45.6

Block 3 – Health Services		

Outpatient follow-up location		
Private service or insurance plan	658	38.1
Public network	1,292	61.9
Type of delivery		
Normal	1,092	44.7
Cesarean	1,256	55.3

LEVEL 2. Contextual factors		

Municipal prevalence of child undernutrition^c^ (%)		
≥ 10	416	14.6
< 10	1,954	85.4
Municipality HDI		
Low (0.500–0.599) + Medium (0.600–0.699)	307	3.8
High (0.700–0.799)	1,593	53.2
Very high (> 0.800)	470	43.0

General feeding characteristics	N	Yes (%)

Received breastfeeding the previous day^d^	1,317	54.5
Consumption of liquids the day before		
Water	2,135	89.2
Tea	507	21.5
Natural juice	1,579	70.8
Non-breast milk	1,700	71.5
Milk^e^	1944	80.9
Coffee	183	7.4
Soda	182	8.1
Industrialized juice	300	11.9
Consumption of semisolid foods the day before		
Meat	1,465	77.7
Bean	1,368	67.3
Vegetables	1,788	93.5
Fruits	1,675	74.1
Porridge	1,262	49.3
Sweetened preparations^f^	1,192	49.2
Crackers or crisps	1,341	53.9
Main meal (lunch and/or dinner)	1,936	84.5
Feeding quality		
Ultra-processed foods^g^	1,439	58.9
Dietary diversity^h^	715	28.5

HDI: human development index

^a ^Median

^b ^Interquartile range

^c ^Estimated from the 2006 National Demographic and Health Survey data.

^d ^If the LBW infant aged 6 months and 0 days to 11 months and 29 days received breastfeeding on the day before the survey.

^e ^Consumption of breast milk, other types of milk or porridge.

^f ^Foods sweetened with sugar, honey, molasses or sweetener.

^g ^Consumption of any of the following foods: soda, ultra-processed juice and cookies, crackers or crisps.

^h ^Consumption of five food groups in the last 24 hours: meat, beans, vegetables, fruits and milk (breast or other).

Table 2 shows the results of the analysis adjusted only for infant age for both outcomes. Regarding dietary diversity, its prevalence was higher among infants whose mothers had 12 or more years of schooling and did not work outside the home. The same was true for those living in municipalities with child undernutrition prevalence below 10%. Ultra-processed foods were most consumed by infants whose mothers had between 9 and 12 years of schooling, were under 20 years of age, were multiparous and lived in municipalities with a prevalence of child undernutrition below 10%.

**Table 2:** Factors associated with dietary diversity and consumption of ultra-processed foods and their prevalence ratios (PR) adjusted by the age of infants with low birth weight aged 6 to 11 months and 29 days. Breastfeeding Prevalence Survey in Brazilian Municipalities, 2008.

Variable	Dietary diversity					Ultra-processed foods				

	Chi-square test			Poisson Regression		Chi-square test			Poisson Regression	

	N_total_	Yes (%)	p	PR^a^ (95%CI)	p	N_total_	Yes (%)	p	PR^a^ (95%CI)	p
LEVEL 1. Infants, maternal, socioeconomic and health service characteristics										

Block 1 – Socioeconomic characteristic										

Maternal education (years)										
≤ 8	831	25.9	**0.003**	1		829	62.6	**0.064**	1	
9–12	890	27.2		1.05 (0.92–1.21)	**0,001** ^**b**^	887	59.2		0.95 (0.88–1.03)	**0.046** ^**b**^
≥ 12	248	37.5		1.49 (1.25–1.78)		246	52.3		0.85 (0.72–1.01)	

Block 2 – Infants and maternal characteristics										

Sex										
Male	1,078	24.2	**0.114**	1		1,073	56.9	0.218	1	
Female	1,291	31.9		1.27 (0.93–1.73)	**0,128**	1,288	60.6		1.04 (0.95–1.14)	0.425
Maternal age range (years)										
< 20	384	25.8		1		383	61.4		1	
20–35	1,339	27.8	0.651	1.05 (0.88–1.24)	0,526^b^	1,333	60.6	0.190	0.98 (0.90–1.08)	0.047^b^
≥ 35	259	30.3		1.13 (0.77–1.66)		259	54.6		0.86 (0.74–1.00)	
Maternal work										
Does not work outside home	1,381	25.0	**< 0.001**	1		1,377	59.3	0.320	1	
Works outside home	514	35.5		1.42 (1.25–1.61)	**< 0,001**	511	61.5		1.03 (0.96–1.11)	0.450
Parity										
Primiparous	1,058	28.0	0.847	1		1,055	57.4	**0.007**	1	
Multiparous	886	27.5		1.00 (0.84–1.18)	0,967	883	63.6		1.11 (1.02–1.22)	**0.016**

Block 3 – Health services										

Outpatient follow-up location										
Private service or insurance plan	658	29.5	0.923	1		656	55.9	0.290	1	
Public network	1,291	29.8		1.05 (0.77–1.43)	0,736	1,285	61.4		1.14 (1.00–1.30)	**0.052**
Type of delivery										
Normal	1,092	26.3	**0.037**	1		1,090	59.9	0.342	1	
Cesarean	1,256	30.5		1.15 (0.99–1.33)	**0,067**	1,250	58.1		0.96 (0.89–1.03)	0.249

LEVEL 2. Contextual factors										

Municipal prevalence of child undernutrition (%)										
≥ 10	416	16.7	**< 0.001**	1		414	50.0	**0.005**	1	
< 10	1,953	30.6		1.66 (1.23–2.23)	**0,001**	1,947	60.5		1.17 (1.04–1.31)	**0.011**
Municipality HDI										
Low (0.500–0.599) + Medium (0.600–0.699)	307	24.7		1		304	64.9		1	
High (0.700–0.799)	1,592	27.4	0.548	1.13 (0.83–1.54)	0,641^b^	1,588	56.4	0.280	0.87 (0.80–0.94)	0.604^b^
Very high (> 0.800)	470	30.2		1.19 (0.79–1.80)		469	61.5		0.93 (0.78–1.11)	

HDI: human development index

^a ^PR values adjusted for infants’ age.

^b ^p of linear trend.

p of linear trend of infant age (in months) for diversified diet and ultra-processed foods: <0.001.

P values < 0.15 shown in bold

Tables 3 and 4 show the results of the multilevel analysis for the outcomes dietary diversity and UPF consumption, respectively. Even after adjusting for other variables, dietary diversity was more prevalent in LBW infants whose mothers had 12 years or more of schooling (PR = 1.35; 95%CI 1.16–1.58), worked outside the home (PR = 1.28; 95%CI 1.11–1.48) and lived in municipalities with a prevalence of child undernutrition below 10% (PR = 1.66; 95%CI 1.23–2.24). UPF showed results similar to those of the “crude” analysis: the prevalence of consumption was higher among infants whose mothers were under 20 years old (PR = 1.31; 95%CI 1.12–1.52), were multiparous (PR = 1.17; 95%CI 1.03–1.30) and lived in municipalities with a prevalence of child undernutrition below 10% (PR = 1.17; 95%CI 1.04–1.31).

**Table 3:** Individual factors and contextual factor of dietary diversity and adjusted prevalence ratios (PR) of low birth weight infants aged 6 to 11 months and 29 days in 64 Brazilian municipalities. Breastfeeding Prevalence Survey in Brazilian municipalities, 2008.

Variable	Template 0 (n = 2,369)	Template 1 (n = 2,369)	Template 2 (n = 1,877)	Template 3 (n=1,866)
			
	PR (95%CI)	PR (95%CI)	PR (95%CI)	PR (95%CI)
Fixed Effects – Constant	0.07 (0.04–0.13)	0.05 (0.03–0.08)	0.03 (0.02–0.06)	0.03 (0.01–0.06)

LEVEL 1. Infants, maternal, socioeconomic and health service characteristic				

Block 1 – Socioeconomic characteristics				

Maternal education (year)				
≤ 8			1	1
9–12			0.99 (0.86–1.13)	0.99 (0.87–1.14)
≥ 12			1.37 (1.18–1.60)	1.35 (1.16–1.58)
p			0.005*	0.007*

Block 2 – Maternal characteristic				

Maternal work				
Does not work outside home			1	1
Works outside home			1.29 (1.12–1.49)	1.28 (1.11–1.48)
p			**0.001**	**0.001**

Block 3 – Health services				

Type of delivery				
Normal				1
Cesarean				1.08 (0.94–1.24)
p				0.266

LEVEL 2. Contextual factor				

Municipal prevalence of child undernutrition (%)				
≥ 10		1	1	1
< 10		1.66 (1.23–2.24)	1.68 (1.25–2.25)	1.66 (1.23–2.24)
p		**0.001**	**0.001**	**0.001**

Random Effect – Municipalities – Constant	0.03 (0.01–0.11)	0.01 (0.0005–0.18)	1.92e^-32^ (3.37e^-36^–1.10e^-28^)	3.20e^-34^ (1.87e^-38^–5.46e^-30^)

Variance (-2loglikelihood)	3,038.2424	3,013.0438	2,310.959	2,295.2214

Template 0: Age + sex of infants

Template 1: Template 0 + municipal prevalence of child undernutrition

Template 2: Template 1 + maternal education and work

Template 3: Template 2 + type of delivery

* p of linear trend

P values < 0.05 shown in bold

**Table 4:** Individual factors and contextual factor of ultra-processed food intake and adjusted prevalence ratios (PR) of low birth weight infants aged 6 to 11 months and 29 days in 64 Brazilian municipalities. Breastfeeding Prevalence Survey in Brazilian Municipalities, 2008.

Variable	Template 0 (n = 2,361)	Template 1 (n = 2,361)	Template 2 (n = 1,915)	Template 3 (n = 1,564)
			
	PR (95%CI)	PR (95%CI)	PR (95%CI)	PR (95%CI)
Fixed Effects – Constant	0.33 (0.27–0.39)	0.29 (0.24–0.34)	0.20 (0.17–0.24)	0.19 (0.15–0.24)

LEVEL 1. Infants, maternal, socioeconomic and health services characteristics				

Block 1 – Socioeconomic characteristic				

Maternal education (years)				
≥ 12			1	1
9–12			1.06 (0.89–1.26)	1.09 (0.88–1.34)
≤ 8			1.10 (0.89–1.36)	1.04 (0.82–1.32)
p			0.358*	0.974

Block 2 – Maternal characteristics				

Maternal age range (years)				
≥ 35			1	1
20–35			1.19 (1.09–1.29)	1.17 (1.06–1.30)
< 20			1.30 (1.15–1.46)	1.31 (1.12–1.52)
p			< 0.001*	**0.001**
Parity				
Primiparous			1	1
Multiparous			1.16 (1.03–1.30)	1.17 (1.04–1.31)
p			**0.012**	**0.009**

Block 3 – Health services				

Outpatient follow-up location				
Private service or insurance plan				1
Public network				1.06 (0.94–1.21)
p				0.345

LEVEL 2. Contextual factor				

Municipal prevalence of child undernutrition (%)				
≥ 10		1	1	1
< 10		1.17 (1.04–1.31)	1.26 (1.13–1.42)	1.25 (1.08–1.44)
p		**0.011**	**< 0.000**	**0.003**

Random Effect – Municipalities – Constant	7.73e^-36^ (3.92e^-36^–1.53e^-35^)	6.69e^-35^ (8.28e^-36^–5.41e^-34^)	1.03e^-35^ (5.23e^-36^–2.05e^-35^)	3.90e^-34^ (1.84e^-35^–8.29e^-33^)

Variance (-2loglikelihood)	4,193.0474	4,191.4236	3,385.5084	2,758.0814

Template 0: Age + sex of infants

Template 1: Template 0 + municipal prevalence of child undernutrition

Template 2: Template 1 + maternal education, maternal age and parity

Template 3: Template 2 + outpatient follow-up location

* p of linear trend

P values < 0.05 shown in bold

## DISCUSSION

This study presents, in an unprecedented way, the complementary feeding of LBW Brazilian children, evaluated by indicators of two major dimensions of interest in the feeding of vulnerable populations: the positive dimension, related to dietary diversity, and the negative dimension, related to consumption of UPF. Only 28.5% of the LBW infants in the 64 municipalities studied consumed the five food groups on the day before the survey, with dietary diversity below the recommended. More than half of infants (58.9%) consumed UPF, a practice considered inappropriate not only because of the negative nutritional profile of these foods, but also because they usually replace non- or minimally processed foods. The low prevalence of a diverse diet coupled with the high prevalence of UPF consumption characterizes the low quality of LBW Brazilian infants’ diet. Although more than half of infants were breastfed between 6 and 12 months, a rate higher than the national average, complementary feeding contradicts recommendations, increasing the risk of obesity and future chronic diseases.

The health harms indicated by these findings are numerous. UPF have high energy density (high concentration of sugar and saturated and trans fat) and low density of proteins, fibers and most micronutrients, including zinc^24^, and are nutritionally inadequate for infants – especially those born with low weight, for their increased nutritional needs. Due to the small gastric capacity of infants, UPF eventually replace appropriate foods and age-appropriate culinary preparations, causing a decrease in dietary diversity. By stimulating excessive consumption, UPF impact negatively the hunger-satiety self-regulation mechanism, which can be extremely harmful for breastfed infants. In addition, the safety of CF is compromised with the offer of UPF due to the presence of additives in these foods. Finally, due to the importance of the infant’s familiarization with a healthy eating environment in this crucial phase of habit formation, exposure to UPF can have deleterious effects in the short and long term.

Considering the similarity of the methodology used, the prevalence of dietary diversity (28.5%) among LBW infants in these municipalities was less favorable than the results found in the cross-sectional study conducted in Barra Mansa (RJ) on the day of the National Vaccination Campaign in 2006, with children from the general population. The study showed that 35.5% of the children received diversified food in the second semester of life^20^.

The high consumption of UPF (58.9%) in the studied population seems to accompany the important changes observed in the eating habits of the Brazilian population in recent decades, marked by decreased consumption of traditional foods such as rice and beans and increased consumption of UPF^24^.Soft drinks, ultra-processed juices, cookies and ready-to-eat crisps, among others, are present in high frequency in the feeding of children under two years of age^7 , 17 , 25 , 26^, and those born with low weight seem not to be protected from the problem.

The analysis of factors associated with dietary diversity showed that maternal education and work and infant’s region of residence (with a higher or lower prevalence of child undernutrition) determine whether or not LBW children have access to healthy eating. LBW infants’ food intake also varied according to the socioeconomic status of the families. LBW infants whose mothers had eight years or less of schooling and did not work outside the home had lower dietary diversity. The negative association between maternal education and diet quality, characterized by high UPF consumption and low diet adequacy^27^, as well as between maternal work and inappropriate eating practices, is consistent with findings in countries in Africa, Asia and Latin America, including Brazil^28^.

Maternal education lost statistical significance in the multiple analysis for UPF consumption. However, Saldiva et al.^17^found that, in Brazil, children living in the capitals whose mothers had no education were two to three times more likely to consume ultra-processed juices, soft drinks and sweetened foods than children of mothers with higher education.

Regarding maternal age, other studies have also found an association between young mothers and the provision of foods not recommended for infants younger than one year, such as industrialized foods, as a substitute for culinary preparations^29^. This speaks in favor of other factors influencing the maternal choice of offering ready-made or ready-to-eat foods, such as low maternal confidence in her own culinary skills or lack of interest in acquiring them because they are unaware of the importance of this practice for health, and offering their children a diet similar to their own, as observed by Robinson et al.^31^when studying the behavior of teenage mothers, an age group that tends to be the largest consumer of UPF^32^.

UPF consumption was more prevalent among infants born to multiparous women. Studies have found a positive dose-response relationship regarding increased unhealthy eating scores with infant birth order, increased parity, and higher number of siblings^29^. Possibly, this result is explained by the fact that the priorities and dietary needs of older children compete with those of younger ones^31^, and the presence of older children at home reduces the likelihood of preparing a specific feeding for the infant^29^.

Despite the effect of individual variables, the contextual variable referring to 10% or higher prevalence of childhood undernutrition was associated with poorer quality of food in both domains: dietary diversity and UPF consumption. This confirms the important influence of the families’ socioeconomic context on the quality of infant feeding. Municipalities with low socioeconomic conditions, which are concentrated in the North and Northeast, should be a priority in the public policy agenda regarding the direction of educational nutritional actions on food consumption in the first year of life. In addition, the adoption of regulatory measures on the relative price of food, such as taxation of high energy density foods and reduction of the price of fresh or minimally processed foods, such as fruits and vegetables^33 , 34^, would contribute positively to change the feeding of infants living in these places.

Among the limitations of the present study, we highlight the fact that information on infant feeding refers only to the day before the survey, making it impossible to assess the usual food intake. However, the use of a single reminder does not diminish the validity of the study, as the aim was to do group evaluation. Another limitation is the lack of information on gestational age to assess prematurity; however, sensitivity analysis was performed for both outcomes, including only infants born weighing between 2,000 g and 2,499 g and older than six months, with results similar to the total population weighing less than 2,500 g. The similarity of magnitude for the three birth weight strata allowed the inclusion of all infants with LBW in the sample, indicating that they did not differ statistically from preterm infants. In addition, the dietary diversity indicator based on the previous day’s consumption may have led to an underestimation or overestimation of the individuals’ classification and possibly an underestimation of the association between sociodemographic determinants and dietary diversity. Finally, there is a lack of data to quantify the percentage participation of UPF in the total energy intake. On the other hand, research during vaccination campaigns makes it possible to obtain information in a short period and at low cost, and the use of multilevel analysis allows to obtain estimates that take into account the hierarchical level of data and intragroup correlation. It is also noteworthy that BPS is the last epidemiological study of population representativeness conducted in Brazil that allows to evaluate the food intake of LBW infants under one year.

In the Brazilian context, this is the first study to analyze factors associated with the feeding quality of LBW infants, taking into account both dietary diversity and exposure to UPF. Our findings indicate that individual and contextual factors impact the food quality of this vulnerable population, suggesting the need for effective strategies to increase the consumption of fresh or minimally processed foods and reduce the consumption of ultra-processed foods. In addition, monitoring these practices is important to identify the impact of public policies aimed at healthy CF and for at-risk groups to receive more attention from health services.

## Footnotes

a UNICEF Data. Low birth weight. New York; 2019 [cited Feb 5, 2018]. Available from: https://data.unicef.org/topic/nutrition/low-birthweight/

b Programa das Nações Unidas para o Desenvolvimento; Instituto de Pesquisa Econômica Aplicada; Fundação João Pinheiro. Consulta. Brasília, DF: PNUD; IPEA; Fundação João Pinheiro; 2013 [cited on Mar 01, 2018]. Available from: http://www.atlasbrasil.org.br/2013/pt/consulta/
