# Supplementary material for: Feeding practices of low birth weight Brazilian infants and associated factors
Source: Rev Saude Publica. 2020 Jan 23;54:14. doi: 10.11606/s1518-8787.2020054001028 (PMC6986864; doi:10.11606/s1518-8787.2020054001028)
Supplement: Translation

# Translation: Práticas alimentares de lactentes brasileiros nascidos com baixo peso e fatores associados

**Authors:** Naiá Ortelan, Daniela Almeida Neri, Maria Helena D’Aquino Benicio

**Affiliations:** I Brasil Universidade de São Paulo. Faculdade de Saúde Pública. Departamento de Nutrição em Saúde Pública. São Paulo, SP, Brasil.; II Brasil Universidade de São Paulo. Faculdade de Saúde Pública. Núcleo de Pesquisas Epidemiológicas em Nutrição e Saúde (NUPENS). São Paulo, SP, Brasil.

**Keywords:** Recém-Nascido de Baixo Peso, Alimentos Infantis, Nutrição do Lactente, Alimentos, Dieta e Nutrição, Alimentos Ultraprocessados

## Abstract

**OBJETIVO:**

Caracterizar a alimentação complementar e analisar a influência de fatores individuais e contextuais sobre práticas alimentares de lactentes que nasceram com baixo peso.

**MÉTODOS:**

Este estudo transversal incluiu 2.370 lactentes nascidos com baixo peso e com idade entre 6 e 12 meses incluídos na Pesquisa de Prevalência de Aleitamento Materno em Municípios Brasileiros (2008), que abrangeu as 26 capitais, o Distrito Federal e mais 37 municípios. As práticas alimentares foram avaliadas usando dois indicadores: i) diversidade alimentar, caracterizada pelo consumo dos cinco grupos alimentares: carnes, feijão, legumes e verduras, frutas e leite; ii) consumo de alimentos ultraprocessados, caracterizado pela ingestão de pelo menos um dos seguintes alimentos no dia anterior à pesquisa: refrigerante, ou suco industrializado, ou bolacha, biscoito e salgadinho. As covariáveis de interesse corresponderam às características socioeconômicas, dos lactentes, das mães e dos serviços de saúde. O fator contextual foi a “prevalência municipal de desnutrição infantil”. O efeito individualizado dos fatores de estudo sobre os desfechos foi avaliado mediante regressão de Poisson com estrutura multinível.

**RESULTADOS:**

Aproximadamente 59% dos lactentes consumiram alimentos ultraprocessados, enquanto 29% apresentaram diversidade alimentar. Mães que residiam em municípios com prevalência de desnutrição infantil inferior a 10%, com maior nível de escolaridade e que trabalhavam fora de casa foram mais propensas a oferecer diversidade alimentar. O consumo de alimentos ultraprocessados foi maior entre lactentes residentes em municípios com prevalência de desnutrição infantil inferior a 10%, cujas mães eram mais jovens e multíparas.

**CONCLUSÕES:**

A baixa prevalência de alimentação diversa aliada à alta prevalência do consumo de alimentos ultraprocessados caracteriza a baixa qualidade da alimentação dos lactentes brasileiros com baixo peso ao nascer. Fatores individuais e contextuais impactam a qualidade da alimentação dessa população, sugerindo a necessidade de adoção de estratégias eficazes para aumentar o consumo de alimentos *in natura* e minimamente processados e diminuir o consumo de alimentos ultraprocessados por esta população vulnerável.

## INTRODUÇÃO

O baixo peso ao nascer (BPN, < 2.500 g) é considerado um importante problema de saúde pública global por estar associado a um alto índice de morbimortalidade, risco de crescimento inadequado e carências específicas, bem como ganho de peso rápido, além de problemas cognitivos e comportamentais ao longo da vida^1^. Apesar dos grandes avanços no cuidado pré-natal nos últimos anos, sua prevalência se mantém próxima a 8% desde o ano 2000 no Brasil, e a incidência mundial se manteve em 15% entre 2008–2012^a^.

O BPN ocorre como consequência da prematuridade (idade gestacional inferior a 37 semanas), da restrição de crescimento intrauterino (RCIU) ou da associação de ambas, estando relacionado, principalmente, a condições de pobreza, má nutrição e alimentação insuficiente^2 , 3^. Quando associado ao RCIU, o BPN torna as crianças muito vulneráveis a desvios nutricionais, tanto de escassez quanto de excesso. Estudos epidemiológicos e clínicos em crianças pequenas para a idade gestacional observaram uma forte associação entre agravos ocorridos na vida fetal (escassez de nutrientes) ou nas fases iniciais da vida extrauterina (recuperação nutricional rápida em fase precoce da vida pós-natal) e o surgimento de doenças crônicas não transmissíveis (DCNT) ao longo da vida, tais como obesidade, hipertensão, doença cardiovascular, resistência à insulina e diabetes tipo 2^4^.

Embora não haja consenso sobre as necessidades nutricionais dos lactentes que nascem com baixo peso, sabe-se que, uma vez que alcancem a alimentação completa e apresentem ganho de peso adequado para alta hospitalar, as recomendações nutricionais e o acompanhamento de saúde tornam-se semelhantes aos da população geral. Assim, pressupõe-se que, durante os primeiros dois anos de vida, as crianças com BPN estão expostas à mesma situação de alimentação e nutrição dos menores de dois anos da população geral brasileira, num cenário marcado por: introdução precoce da alimentação complementar (AC)^7^, baixo consumo de refeições apropriadas em consistência e textura para a idade e elevado consumo de alimentos ultraprocessados (AUP)^7^. Esses alimentos são formulações industriais prontas para o consumo, feitas inteiramente ou majoritariamente de substâncias extraídas de alimentos (óleos, gorduras, açúcar, proteínas), derivadas de constituintes de alimentos (gorduras hidrogenadas, amido modificado) ou sintetizadas em laboratório com base em matérias orgânicas (corantes, aromatizantes, realçadores de sabor e outros aditivos usados para alterar propriedades sensoriais)^8^, por exemplo, refrigerantes e outras bebidas adoçadas, biscoitos recheados, salgadinhos, macarrão instantâneo, sorvetes, guloseimas, cereais matinais açucarados, barras de cereal, embutidos, salsichas, hambúrgueres (não artesanais) e pães de forma, de hambúrguer, de cachorro-quente, entre outros.

A promoção da alimentação complementar adequada e saudável tem inúmeros benefícios: é considerada a terceira ação mais efetiva com potencial de prevenir 6% de todas as mortes de menores de cinco anos no mundo^9^; está associada ao estabelecimento de hábitos alimentares saudáveis, que se refletem na infância^10 , 11^e na vida adulta^12^; está entre os fatores de risco modificáveis para a obesidade em crianças^13^; contribui para que as crianças alcancem seu potencial de desenvolvimento e se tornem adultos saudáveis, com maior capacidade intelectual e produtiva^14^; favorece o desenvolvimento econômico sustentável e a redução da pobreza de uma nação^15^.

Assim, é essencial monitorar se a alimentação das crianças com BPN atende às suas necessidades nutricionais e reduz o risco de doenças crônicas. Nessa perspectiva, em 1999 realizou-se o primeiro inquérito denominado Pesquisa de Prevalência de Aleitamento Materno (PPAM) nas Capitais Brasileiras e Distrito Federal^16^durante a campanha nacional de vacinação e, em 2008, a II PPAM^7^, justificada pelos gestores pela necessidade de uma análise da evolução das práticas alimentares de crianças menores de 1 ano, tendo em vista as diversas ações desenvolvidas no âmbito da política nacional. Porém, até o momento não há conhecimento sobre essas práticas alimentares entre a população de lactentes nascidos com baixo peso. Esses dados também são escassos na literatura internacional.

Visando diferenciar esse estudo dos demais, definiu-se uma população de estudo e não uma variável, destacando a originalidade centrada na ênfase ao BPN em vez de conduzir mais um trabalho de prevalência “BPN versus não BPN”. Dessa forma, foi possível explorar o perfil e os diferenciais dessa população vulnerável, ressaltando sua importância. Tendo em vista a dupla carga de riscos nutricionais a que as crianças brasileiras com BPN estão expostas, a heterogeneidade da renda e escolaridade no Brasil, a necessidade de monitoramento dessa população e a disponibilidade de dados do inquérito da II PPAM, o presente trabalho teve como objetivo caracterizar a AC de lactentes brasileiros nascidos com baixo peso e com idade de seis meses e 0 dias a 11 meses e 29 dias (ou seja, de seis meses completos a doze incompletos) e analisar a influência de fatores individuais e contextuais sobre práticas alimentares relacionadas à diversidade da alimentação e ao consumo de alimentos ultraprocessados. Os resultados poderão subsidiar e contribuir de forma relevante para o aprimoramento de políticas públicas voltadas à melhoria da alimentação infantil nesse grupo.

## MÉTODOS

Este estudo transversal é parte da PPAM em Municípios Brasileiros (2008), realizada nas 26 capitais brasileiras, Distrito Federal e outros 37 municípios, com população de crianças menores de um ano superior a 4 mil, com o objetivo de avaliar a situação da amamentação e da AC no país. Os dados foram obtidos durante a Campanha Nacional de Vacinação de 2008, utilizando um questionário fechado que incluía questões sobre características sociodemográficas das mães ou responsáveis e das crianças, além do consumo de leite materno, outros tipos de leite e outros alimentos no dia anterior à pesquisa. Detalhes sobre o cálculo do tamanho amostral e sobre a coleta de dados podem ser encontrados em outras publicações^7 , 17^.

No presente estudo, foram incluídos os 2.370 lactentes nascidos com baixo peso e com idade de seis meses e 0 dias a 11 meses e 29 dias (devido à recomendação de amamentação exclusiva até os seis meses) residentes em 64 municípios brasileiros. A definição da população deste artigo pode ser consultada no anexo ( Figura 1 ).

**Figura 1:** Fluxograma que representa as definições da população deste estudo. PN: peso ao nascer

Por se tratar de uma pesquisa feita com amostragem probabilística complexa, foram utilizados procedimentos específicos para sua análise. Devido às diferenças populacionais entre os municípios estudados, cada plano correspondeu a uma fração amostral diferente, representada pelo tamanho estimado da amostra sobre o número de crianças a serem vacinadas. O inverso dessa fração foi aplicado como peso das crianças em cada município^7^.

Avaliamos duas dimensões de interesse na alimentação de populações vulneráveis: i) a fração composta por alimentos não ou minimamente processados, que se relaciona positivamente com a adequação nutricional (dimensão positiva); ii) a fração composta por AUP, que se relaciona positivamente com alta densidade energética, excesso de açúcar livre e de gordura saturada e *trans* , e inadequação de proteínas e micronutrientes (dimensão negativa).

Para a avaliação da diversidade alimentar, utilizou-se um indicador adaptado ao proposto pela Organização Mundial da Saúde (OMS)^18^. No presente estudo, a diversidade foi caracterizada como o consumo de cinco grupos de alimentos nas últimas 24 horas: carnes, feijão, legumes ou verduras, frutas e leite (materno ou outro), conforme proposto em outros estudos^19 , 20^.

A avaliação do consumo de AUP foi baseada na classificação NOVA, que categoriza os alimentos de acordo com a extensão e o propósito de seu processamento^21^, e no conjunto de alimentos considerados ultraprocessados no questionário da PPAM: refrigerante, suco industrializado e bolacha, biscoito e salgadinho.

Assim, os desfechos do presente estudo foram: i) “diversidade alimentar”, definida como o consumo de cinco grupos de alimentos pelo menos uma vez no dia anterior à pesquisa, e ii) “consumo de AUP”, quando o lactente consumiu, no dia anterior à pesquisa, pelo menos um dos alimentos mencionados anteriormente. Ambas as variáveis dependentes foram dicotomizadas em 0 (não) e 1 (sim).

As covariáveis de interesse corresponderam às características socioeconômicas (representadas pela *proxy* escolaridade materna em anos de estudo: ≤ 8, 9|–12, ≥ 12), dos lactentes (idade: 6|–7 meses, 7|–8 meses, 8|–9 meses, 9|–10 meses, 10|–11 meses, 11|–12 meses; sexo: masculino, feminino), das mães (faixa etária em anos: < 20, 20|–35, ≥ 35; trabalha fora: não, sim; paridade: primípara, multípara) e dos serviços de saúde (local de acompanhamento ambulatorial: serviço particular ou por convênio, rede pública; tipo de parto: normal, cesárea). O primeiro fator contextual estudado referente ao município foi a prevalência municipal de desnutrição infantil (≥ 10%, < 10%), variável utilizada como *proxy* de pobreza e estimada por Benicio et al.^22^a partir de dados da Pesquisa Nacional de Demografia e Saúde (PNDS) de 2006 e da amostra do Censo Demográfico de 2000. A desnutrição infantil foi aferida pelo déficit de estatura para a idade inferior a -2 escores Z do padrão de crescimento da OMS de 2006. As estimativas de prevalência da desnutrição infantil foram produzidas para cada um dos 5.507 municípios brasileiros existentes em 2000. O método empregado para a obtenção dessas estimativas baseou-se no desenvolvimento de modelos estatísticos individuais de predição, utilizando análise multinível com base na PNDS de 2006, com inclusão de variáveis preditivas aferidas de forma semelhante nas duas pesquisas. A prevalência foi então estimada pela média da probabilidade individual das crianças residentes em cada município estudado pela amostra do Censo de 2000. O segundo fator contextual foi o índice de desenvolvimento humano (IDH) do município, utilizado como indicador direto para referir a condição socioeconômica dos 64 municípios estudados em 2010^b^, estratificado em ordem crescente de classificação: baixo (0,500–0,599) + médio (0,600–0,699), alto (0,700–0,799), muito alto (> 0,800).

O modelo conceitual utilizado está representado na Figura 2 . Nele, observa-se que a estratégia de modelagem hierarquizada foi utilizada para incluir as variáveis individuais no modelo. Nessa estratégia, a hierarquização das variáveis independentes é estabelecida num marco conceitual, e a escolha de critérios para selecioná-las requer conhecimentos sobre precedência temporal e determinantes biológicos e sociais, não considerando somente o aspecto estatístico. A hierarquização foi mantida durante toda a análise, possibilitando a seleção das variáveis mais fortemente associadas aos desfechos de interesse^23^.

**Figura 2:** Marco conceitual para investigação dos fatores individuais e contextuais da diversidade alimentar e do consumo de alimentos ultraprocessados. IDH: índice de desenvolvimento humano

A associação entre as variáveis independentes e os desfechos foi avaliada inicialmente utilizando-se regressão de Poisson com ajuste apenas pela idade do lactente. Assim, foram selecionadas àquelas que poderiam influenciar os desfechos. O efeito dessas, livre de confusão gerada pelas demais, foi avaliado por regressão de Poisson com estrutura multinível, utilizada em função da organização hierárquica da população em relação às características socioeconômicas, dos lactentes, maternas e dos serviços de saúde (Nível 1), considerando o contexto (Nível 2), e a existência de correlação intragrupo.

No modelo final, permaneceram como variáveis de ajuste apenas àquelas que apresentaram p < 0,15 na análise ajustada pela idade do lactente. A seleção das variáveis que compuseram o modelo final seguiu o processo de seleção *backward* : no primeiro estágio da modelagem, foram inseridas as variáveis contextuais ajustadas pela idade e sexo do lactente para ambos os desfechos (Nível 2); nos três estágios seguintes, àquelas relacionadas às características socioeconômicas (Bloco 1) ou maternas (Bloco 2) e aos serviços de saúde (Bloco 3), todas do Nível 1. Covariáveis com mais de duas categorias foram introduzidas no modelo como variáveis qualitativas ordinais para estimar o valor de p de tendência linear. Foram apresentados valores de razão de prevalências (RP) e seus respectivos intervalos com 95% de confiança (IC95%). A associação entre o fator de estudo e o desfecho foi considerada significante quando p < 0,05. A avaliação da qualidade de ajuste do modelo multinível foi verificada pelo teste de -2 *loglikelihood* . Todas as análises foram realizadas com o *software* Stata/SE 14.1.

Este projeto foi aprovado pelo Comitê de Ética em Pesquisa da Faculdade de Saúde Pública da Universidade de São Paulo, sob parecer número 58147216.1.0000.5421.

## RESULTADOS

A Tabela 1 mostra as características da amostra e da alimentação dos lactentes estudados. A mediana da idade dos lactentes foi 8,9 meses e do peso ao nascer, 2.230 g, sendo que 72% nasceram com peso entre 2.000 e 2.499 g. Cerca de 67% das mães tinham entre 20 e 35 anos, 71% não trabalhavam fora e 54% eram primíparas. A rede pública foi o principal local de acompanhamento ambulatorial (61,9%) e a cesárea foi o parto mais prevalente (55,3%). Aproximadamente 85% dos lactentes residiam em municípios com prevalência de desnutrição infantil inferior a 10%, e 53% em municípios com alto IDH. A prevalência de aleitamento materno (AM) no dia anterior à pesquisa foi 54,5%. Em relação ao consumo de líquidos no dia anterior à pesquisa, a maior prevalência encontrada foi a de água (89,2%), seguida por leite materno, não materno ou mingau (80,9%), outro tipo de leite que não materno (71,5%) e suco natural (70,8%). Assim, a probabilidade de o lactente receber leite materno foi inferior à encontrada para outros leites e mingau. Entre o consumo de alimentos semissólidos no dia anterior à pesquisa, observou-se maior prevalência de legumes e verduras (93,5%), seguidos pelas carnes (77,7%), frutas (74,1%) e feijão (67,3%). Em relação às práticas alimentares, apesar do alto consumo da refeição principal no almoço e/ou jantar (84,5%), observou-se consumo elevado de AUP (58,9%), e somente 28,5% da população estudada recebeu os cinco grupos de alimentos pelo menos uma vez nas últimas 24 horas.

**Tabela 1:** Características da amostra e da alimentação dos lactentes com baixo peso ao nascer de 6 a 11 meses e 29 dias. Pesquisa de Prevalência de Aleitamento Materno em Municípios Brasileiros, 2008 (n = 2.370).

Características da amostra	N_total_ou mediana^a^	% por categoria ou intervalo interquartil^b^
NÍVEL 1. Características dos lactentes, maternas, socioeconômicas e dos serviços de saúde		

Bloco 1 – Característica socioeconômica		

Escolaridade materna (anos)		
≤ 8	831	40,4
9–12	890	47,1
≥ 12	248	12,5

Bloco 2 – Características dos lactentes e maternas		

Sexo		
Masculino	1.079	43,7
Feminino	1.291	56,3
Idade dos lactentes (meses)	8,9^a^	7,3–10,5^b^
Peso ao nascer (g)	2.230^a^	1.960–2.380^b^
< 1.500	250	11,8
1.500–1.999	380	16,2
2.000–2.499	1.740	72,0
Faixa etária materna (anos)		
< 20	384	18,1
20–35	1.339	66,9
≥ 35	259	15,0
Trabalho materno		
Não trabalha fora	1.381	70,7
Trabalha fora	514	29,4
Paridade		
Primípara	1.058	54,4
Multípara	886	45,6

Bloco 3 – Serviços de saúde		

Local de acompanhamento ambulatorial		
Serviço particular ou por convênio	658	38,1
Rede pública	1.292	61,9
Tipo de parto		
Normal	1.092	44,7
Cesárea	1.256	55,3

NÍVEL 2. Fatores contextuais		

Prevalência municipal de desnutrição infantil^c^(%)		
≥ 10	416	14,6
< 10	1.954	85,4
IDH do município		
Baixo (0,500–0,599) + Médio (0,600–0,699)	307	3,8
Alto (0,700–0,799)	1.593	53,2
Muito alto (> 0,800)	470	43,0

Características gerais da alimentação	N	Sim (%)

Recebeu aleitamento materno no dia anterior^d^	1.317	54,5
Consumo de líquidos no dia anterior		
Água	2.135	89,2
Chá	507	21,5
Suco natural	1.579	70,8
Leite não materno	1.700	71,5
Leites^e^	1944	80,9
Café	183	7,4
Refrigerante	182	8,1
Suco industrializado	300	11,9
Consumo de alimentos semissólidos no dia anterior		
Carne	1.465	77,7
Feijão	1.368	67,3
Legumes e verduras	1.788	93,5
Frutas	1.675	74,1
Mingau	1.262	49,3
Preparações adoçadas^f^	1.192	49,2
Bolacha ou salgadinho	1.341	53,9
Refeição principal (almoço e/ou jantar)	1.936	84,5
Qualidade da alimentação		
Alimentos ultraprocessados^g^	1.439	58,9
Diversidade alimentar^h^	715	28,5

IDH: índice de desenvolvimento humano

^a^Mediana

^b^Intervalo interquartil

^c^Estimada a partir de dados da Pesquisa Nacional de Demografia e Saúde de 2006.

^d^Se a criança com BPN de 6 meses e 0 dias a 11 meses e 29 dias recebeu aleitamento materno no dia anterior à pesquisa.

^e^Consumo de leite materno, outros tipos de leite ou mingau.

^f^Alimentos adoçados com açúcar, mel, melado ou adoçante.

^g^Consumo de qualquer um dos seguintes alimentos: refrigerante, suco industrializado e bolacha, biscoito ou salgadinho.

^h^Consumo de cinco grupos alimentares nas últimas 24 horas: carnes, feijão, legumes ou verduras, frutas e leite (materno ou outro).

Os resultados da análise ajustada somente pela idade dos lactentes, para ambos os desfechos, estão descritos na Tabela 2 . Em relação à diversidade alimentar, sua prevalência foi maior entre lactentes cujas mães tinham escolaridade igual ou superior a 12 anos e não trabalhavam fora. O mesmo se verificou entre aqueles que residiam em municípios com prevalência de desnutrição infantil inferior a 10%. Já os alimentos ultraprocessados foram mais consumidos por lactentes cujas mães tinham entre 9 e 12 anos de escolaridade, idade inferior a 20 anos, eram multíparas e residiam em municípios com prevalência de desnutrição infantil inferior a 10%.

**Tabela 2:** Fatores associados à diversidade alimentar e ao consumo de alimentos ultraprocessados e respectivas razões de prevalência (RP) ajustadas pela idade dos lactentes com baixo peso ao nascer de 6 a 11 meses e 29 dias. Pesquisa de Prevalência de Aleitamento Materno em municípios brasileiros, 2008.

Variável	Diversidade alimentar					Alimentos ultraprocessados				

	Teste do qui-quadrado			Regressão de Poisson		Teste do qui-quadrado			Regressão de Poisson	

	N_total_	Sim (%)	p	RP (IC95%)	p	N_total_	Sim (%)	p	RP (IC95%)	p
NÍVEL 1. Características dos lactentes, maternas, socioeconômicas e dos serviços de saúde										

Bloco 1 – Característica socioeconômica										

Escolaridade materna (anos)										
≤ 8	831	25,9	**0,003**	1		829	62,6	**0,064**	1	
9–12	890	27,2		1,05 (0,92–1,21)	**0,001** ^**b**^	887	59,2		0,95 (0,88–1,03)	**0,046** ^**b**^
≥ 12	248	37,5		1,49 (1,25–1,78)		246	52,3		0,85 (0,72–1,01)	

Bloco 2 – Características dos lactentes e maternas										

Sexo										
Masculino	1.078	24,2	**0,114**	1		1.073	56,9	0,218	1	
Feminino	1.291	31,9		1,27 (0,93–1,73)	**0,128**	1.288	60,6		1,04 (0,95–1,14)	0,425
Faixa etária materna (anos)										
< 20	384	25,8		1		383	61,4		1	
20–35	1.339	27,8	0,651	1,05 (0,88–1,24)	0,526^b^	1.333	60,6	0,190	0,98 (0,90–1,08)	0,047^b^
≥ 35	259	30,3		1,13 (0,77–1,66)		259	54,6		0,86 (0,74–1,00)	
Trabalho materno										
Não trabalha fora	1.381	25,0	**< 0,001**	1		1.377	59,3	0,320	1	
Trabalha fora	514	35,5		1,42 (1,25–1,61)	**< 0,001**	511	61,5		1,03 (0,96–1,11)	0,450
Paridade										
Primípara	1.058	28,0	0,847	1		1.055	57,4	**0,007**	1	
Multípara	886	27,5		1,00 (0,84–1,18)	0,967	883	63,6		1,11 (1,02–1,22)	**0,016**

Bloco 3 – Serviços de saúde										

Local de acompanhamento ambulatorial										
Serviço particular ou por convênio	658	29,5	0,923	1		656	55,9	0,290	1	
Rede pública	1.291	29,8		1,05 (0,77–1,43)	0,736	1.285	61,4		1,14 (1,00–1,30)	**0,052**
Tipo de parto										
Normal	1.092	26,3	**0,037**	1		1.090	59,9	0,342	1	
Cesárea	1.256	30,5		1,15 (0,99–1,33)	**0,067**	1.250	58,1		0,96 (0,89–1,03)	0,249

NÍVEL 2. Fatores contextuais										

Prevalência municipal de desnutrição infantil (%)										
≥ 10	416	16,7	**< 0,001**	1		414	50,0	**0,005**	1	
< 10	1.953	30,6		1,66 (1,23–2,23)	**0,001**	1.947	60,5		1,17 (1,04–1,31)	**0,011**
IDH do município										
Baixo (0,500–0,599) + Médio (0,600–0,699)	307	24,7		1		304	64,9		1	
Alto (0,700–0,799)	1.592	27,4	0,548	1,13 (0,83–1,54)	0,641^b^	1.588	56,4	0,280	0,87 (0,80–0,94)	0,604^b^
Muito alto (> 0,800)	470	30,2		1,19 (0,79–1,80)		469	61,5		0,93 (0,78–1,11)	

IDH: índice de desenvolvimento humano

^a^Valores de RP ajustados pela idade das crianças.

^b^p de tendência linear.

p de tendência linear da idade dos lactentes (em meses) para dieta diversificada e alimentos ultraprocessados: < 0,001.

Valores de p < 0,15 apresentados em negrito.

As Tabelas 3 e 4 mostram os resultados da análise multinível para os desfechos diversidade alimentar e consumo de AUP, respectivamente. Mesmo após ajuste para as demais variáveis, a diversidade alimentar foi mais prevalente entre lactentes com BPN cujas mães tinham escolaridade igual ou superior a doze anos (RP = 1,35; IC95% 1,16–1,58), trabalhavam fora (RP = 1,28; IC95% 1,11–1,48) e residiam em municípios com prevalência de desnutrição infantil inferior a 10% (RP = 1,66; IC95% 1,23–2,24). Os AUP apresentaram resultados similares aos da análise “bruta”: a prevalência de consumo foi maior entre lactentes cujas mães tinham idade inferior a 20 anos (RP = 1,31; IC95% 1,12–1,52), eram multíparas (RP = 1,17; IC95% 1,03–1,30) e residiam em municípios com prevalência de desnutrição infantil inferior a 10% (RP = 1,17; IC95% 1,04–1,31).

**Tabela 3:** Fatores individuais e fator contextual da diversidade alimentar e razões de prevalência (RP) ajustadas de lactentes nascidos com baixo peso entre 6 e 11 meses e 29 dias de idade de 64 municípios brasileiros. Pesquisa de Prevalência de Aleitamento Materno em municípios brasileiros, 2008.

Variável	Modelo 0 (n = 2.369)	Modelo 1 (n = 2.369)	Modelo 2 (n = 1.877)	Modelo 3 (n=1.866)
			
	RP_aj_(IC95%)	RP_aj_(IC95%)	RP_aj_(IC95%)	RP_aj_(IC95%)
Efeitos fixos – Constante	0,07 (0,04–0,13)	0,05 (0,03–0,08)	0,03 (0,02–0,06)	0,03 (0,01–0,06)

NÍVEL 1. Características dos lactentes, maternas, socioeconômicas e dos serviços de saúde				

Bloco 1 – Característica socioeconômica				

Escolaridade materna (anos)				
≤ 8			1	1
9–12			0,99 (0,86–1,13)	0,99 (0,87–1,14)
≥ 12			1,37 (1,18–1,60)	1,35 (1,16–1,58)
p			0,005*	0,007*

Bloco 2 – Característica materna				

Trabalho materno				
Não trabalha fora			1	1
Trabalha fora			1,29 (1,12–1,49)	1,28 (1,11–1,48)
p			**0,001**	**0,001**

Bloco 3 – Serviços de saúde				

Tipo de parto				
Normal				1
Cesárea				1,08 (0,94–1,24)
p				0,266

NÍVEL 2. Fator contextual				

Prevalência municipal de desnutrição infantil (%)				
≥ 10		1	1	1
< 10		1,66 (1,23–2,24)	1,68 (1,25–2,25)	1,66 (1,23–2,24)
p		**0,001**	**0,001**	**0,001**

Efeito randômico – Municípios – Constante	0,03 (0,01–0,11)	0,01 (0,0005–0,18)	1,92e^-32^(3,37e^-36^–1,10e^-28^)	3,20e^-34^(1,87e^-38^–5,46e^-30^)

Variância (-2loglikelihood)	3.038,2424	3.013,0438	2.310,959	2.295,2214

Modelo 0: idade + sexo dos lactentes

Modelo 1: Modelo 0 + prevalência municipal de desnutrição infantil

Modelo 2: Modelo 1 + escolaridade e trabalho maternos

Modelo 3: Modelo 2 + tipo de parto

*p de tendência linear

Valores de p < 0,05 apresentados em negrito

**Tabela 4:** Fatores individuais e fator contextual do consumo de alimentos ultraprocessados e razões de prevalência (RP) ajustadas de lactentes nascidos com baixo peso entre 6 e 11 meses e 29 dias de idade de 64 municípios brasileiros. Pesquisa de Prevalência de Aleitamento Materno em municípios brasileiros, 2008.

Variável	Modelo 0 (n = 2.361)	Modelo 1 (n = 2.361)	Modelo 2 (n = 1.915)	Modelo 3 (n = 1.564)
			
	RP_aj_(IC95%)	RP_aj_(IC95%)	RP_aj_(IC95%)	RP_aj_(IC95%)
Efeitos fixos – Constante	0,33 (0,27–0,39)	0,29 (0,24–0,34)	0,20 (0,17–0,24)	0,19 (0,15–0,24)

NÍVEL 1. Características dos lactentes, maternas, socioeconômicas e dos serviços de saúde				

Bloco 1 – Característica socioeconômica				

Escolaridade materna (anos)				
≥ 12			1	1
9–12			1,06 (0,89–1,26)	1,09 (0,88–1,34)
≤ 8			1,10 (0,89–1,36)	1,04 (0,82–1,32)
p			0,358*	0,974

Bloco 2 – Características maternas				

Faixa etária materna (anos)				
≥ 35			1	1
20–35			1,19 (1,09–1,29)	1,17 (1,06–1,30)
< 20			1,30 (1,15–1,46)	1,31 (1,12–1,52)
p			< 0,001*	**0,001**
Paridade				
Primípara			1	1
Multípara			1,16 (1,03–1,30)	1,17 (1,04–1,31)
p			**0,012**	**0,009**

Bloco 3 – Serviços de saúde				

Local de acompanhamento ambulatorial				
Serviço particular ou por convênio				1
Rede pública				1,06 (0,94–1,21)
p				0,345

NÍVEL 2. Fator contextual				

Prevalência municipal de desnutrição infantil (%)				
≥ 10		1	1	1
< 10		1,17 (1,04–1,31)	1,26 (1,13–1,42)	1,25 (1,08–1,44)
p		**0,011**	**< 0,000**	**0,003**

Efeito randômico – Municípios – Constante	7,73e^-36^(3,92e^-36^–1,53e^-35^)	6,69e^-35^(8,28e^-36^–5,41e^-34^)	1,03e^-35^(5,23e^-36^–2,05e^-35^)	3,90e^-34^(1,84e^-35^–8,29e^-33^)

Variância (-2loglikelihood)	4.193,0474	4.191,4236	3.385,5084	2.758,0814

Modelo 0: idade + sexo dos lactentes

Modelo 1: Modelo 0 + prevalência municipal de desnutrição infantil

Modelo 2: Modelo 1 + escolaridade materna, faixa etária materna e paridade

Modelo 3: Modelo 2 + local de acompanhamento ambulatorial

*p de tendência linear

Valores de p < 0,05 apresentados em negrito

## DISCUSSÃO

Este estudo apresenta, de forma inédita, o perfil da alimentação complementar de crianças brasileiras nascidas com baixo peso, avaliada por indicadores de duas grandes dimensões de interesse na alimentação de populações vulneráveis: a dimensão positiva, relacionada à diversidade alimentar, e a negativa, relacionada ao consumo de AUP. Somente 28,5% dos lactentes com BPN dos 64 municípios estudados consumiram os cinco grupos de alimentos no dia anterior à pesquisa, apresentando uma diversidade alimentar aquém do recomendado. Mais da metade dos lactentes (58,9%) consumiram AUP, prática considerada inadequada não apenas pelo perfil nutricional negativo desses alimentos, mas também por normalmente substituírem os alimentos não ou minimamente processados. A baixa prevalência de alimentação diversa aliada à alta prevalência do consumo de AUP caracteriza a baixa qualidade da dieta dos lactentes brasileiros com BPN. Apesar de mais da metade dos lactentes terem sido amamentados entre 6 e 12 meses, taxa superior à média nacional, a alimentação complementar contraria as recomendações, aumentando o risco de obesidade e doenças crônicas futuras.

Os prejuízos desses achados são inúmeros. Os AUP apresentam alta densidade energética (alta concentração de açúcar e gordura saturada e *trans* ) e baixa densidade de proteínas, de fibras e da maioria dos micronutrientes, incluindo o zinco^24^, sendo nutricionalmente inadequados para os lactentes – especialmente os que nascem com baixo peso, por suas necessidades nutricionais aumentadas. Devido à pequena capacidade gástrica dos lactentes, os AUP acabam substituindo alimentos adequados e preparações culinárias apropriadas para a faixa etária, causando prejuízo na diversidade alimentar. Por estimularem o consumo excessivo, especula-se que os AUP impactem negativamente o mecanismo de autorregulação fome-saciedade, o que pode ser extremamente prejudicial para lactentes em aleitamento materno. Além disso, a segurança da AC fica comprometida com a oferta de AUP, pela presença de aditivos característicos destes alimentos. Finalmente, pela importância da familiarização do lactente com um ambiente alimentar saudável nessa fase crucial de formação de hábitos, a exposição à AUP pode causar efeitos deletérios no curto e longo prazos.

Considerando a similaridade da metodologia utilizada, a prevalência da diversidade alimentar (28,5%) entre os lactentes com BPN nesses municípios foi menos favorável que os resultados encontrados no estudo transversal conduzido em Barra Mansa (RJ) no dia da Campanha Nacional de Vacinação de 2006, com crianças da população geral. O estudo mostrou que 35,5% das crianças recebiam alimentação diversificada no segundo semestre de vida^20^.

O consumo elevado de AUP (58,9%) na população estudada parece acompanhar as importantes mudanças observadas nos hábitos alimentares da população brasileira nas últimas décadas, marcadas pela diminuição do consumo de alimentos tradicionais como o arroz e o feijão e aumento do consumo de AUP^24^. Refrigerantes, sucos industrializados, biscoitos e lanches prontos para consumo, entre outros, estão presentes em alta frequência na alimentação de crianças menores de dois anos idade^7 , 17 , 25 , 26^, e os nascidos com baixo peso parecem não estar protegidos do problema.

A análise dos fatores associados à diversidade alimentar mostrou que a escolaridade e trabalho maternos e a região de residência do lactente (com maior ou menor prevalência de desnutrição infantil) determinam se as crianças nascidas com baixo peso têm acesso ou não a uma alimentação saudável. O consumo alimentar dos lactentes com BPN variou também em função da condição socioeconômica das famílias. Lactentes nascidos com baixo peso cujas mães tinham escolaridade igual ou inferior a oito anos e que não trabalhavam fora apresentaram menor diversidade alimentar. A associação negativa entre escolaridade materna e qualidade da dieta, caracterizada pelo alto consumo de AUP e baixa adequação da dieta^27^, bem como entre trabalho materno e práticas alimentares inadequadas, é consistente com achados em países da África, Ásia e América Latina, incluindo o Brasil^28^.

A escolaridade materna perdeu significância estatística na análise múltipla para consumo de AUP. Entretanto, Saldiva et al.^17^verificaram que, no Brasil, crianças residentes nas capitais, filhas de mães sem escolaridade, apresentaram chances duas a três vezes maiores de consumir sucos industrializados, refrigerantes e alimentos adoçados do que filhos de mães com maior escolaridade.

Em relação à faixa etária materna, outros estudos também observaram associação entre mães jovens e oferta de alimentos não recomendados a lactentes menores de um ano, como os alimentos industrializados, em substituição a preparações culinárias^29^. Isso fala a favor de outros fatores influenciarem a escolha materna pela oferta de alimentos prontos ou pré-prontos, tais como a baixa confiança materna no desempenho de suas habilidades culinárias ou a falta de interesse em adquiri-las, por desconhecerem a importância dessa prática para a saúde, e o oferecimento aos filhos de uma alimentação semelhante à própria, como observado por Robinson et al.^31^ao estudar o comportamento de mães adolescentes, grupo etário que tende a ser o maior consumidor de AUP^32^.

O consumo de AUP mostrou-se mais prevalente entre lactentes filhos de mulheres multíparas. Estudos encontraram relação dose-resposta positiva em relação ao aumento de escores de alimentação não saudável com a ordem de nascimento do lactente, com o aumento da paridade e com maior número de irmãos^29^. Possivelmente, esse resultado é explicado pelo fato de que as prioridades e necessidades alimentares dos filhos mais velhos competem com as dos mais novos^31^, além de a presença de crianças mais velhas no domicílio reduzir a probabilidade de preparo de uma alimentação específica para o lactente^29^.

Apesar do efeito das variáveis individuais, a variável contextual referente à prevalência de desnutrição infantil igual ou superior a 10% se associou com a pior qualidade da alimentação nos dois domínios: tanto em relação à diversidade alimentar quanto em relação ao consumo de AUP. Isso confirma a importante influência da condição socioeconômica do contexto em que se inserem as famílias na qualidade da alimentação infantil. Municípios de baixas condições socioeconômicas, os quais se concentram nas regiões Norte e Nordeste, deveriam ser prioridade na agenda de políticas públicas no que se refere ao direcionamento de ações nutricionais educativas sobre consumo alimentar no primeiro ano de vida. Além disso, a adoção de medidas regulatórias sob o preço relativo dos alimentos, como a taxação de alimentos com alta densidade energética e redução do preço de alimentos *in natura* ou minimamente processados, como frutas, legumes e verduras^33 , 34^, contribuiria positivamente para a mudança da alimentação de lactentes residentes nesses locais.

Dentre as limitações do presente estudo, destaca-se o fato das informações referentes à alimentação dos lactentes referirem-se apenas ao dia anterior ao inquérito, impossibilitando avaliar o consumo alimentar habitual. No entanto, a utilização de um único recordatório não diminui a validade do estudo, pois o objetivo foi realizar avaliação de grupo. Outra limitação é a ausência de informações sobre idade gestacional para avaliar prematuridade; contudo, foi realizada análise de sensibilidade para ambos os desfechos, incluindo somente lactentes nascidos com peso entre 2.000 g e 2. 499 g e maiores de seis meses, tendo resultados semelhantes à população total com peso inferior a 2.500 g. A semelhança de magnitude para os três estratos de peso ao nascer permitiu a inclusão de todos os lactentes com BPN da amostra, indicando que esses não diferiram estatisticamente dos prematuros. Ademais, o indicador diversidade alimentar baseado no consumo do dia anterior pode ter levado à sub ou superestimação da classificação dos indivíduos e possivelmente a uma subestimação da associação entre determinantes sociodemográficos e diversidade alimentar. Por último, há a ausência de dados que permitam quantificar a participação percentual de AUP no total calórico. Por outro lado, pesquisas em campanhas de vacinação possibilitam a obtenção de informações em um curto período e com baixo custo, e a utilização da análise multinível permite a obtenção de estimativas que levam em consideração o nível hierárquico dos dados e a correlação intragrupo. Ressalta-se também que a PPAM é o último estudo epidemiológico de representatividade populacional realizado no Brasil que permite avaliar o consumo alimentar de lactentes com BPN menores de um ano.

No contexto brasileiro, este é o primeiro estudo a analisar fatores associados à qualidade da alimentação de lactentes com BPN, levando em consideração tanto a diversidade alimentar quanto a exposição a AUP. Nossos achados indicam que fatores individuais e contextuais impactam a qualidade da alimentação dessa população vulnerável, sugerindo a necessidade de adoção de estratégias eficazes para aumentar o consumo de alimentos *in natura* ou minimamente processados e reduzir o consumo dos ultraprocessados. Além disso, o monitoramento dessas práticas faz-se importante para identificar o impacto das políticas públicas voltadas para a AC saudável e para que grupos de risco recebam mais atenção pelos serviços de saúde.

## Footnotes

a UNICEF Data. Low birth weight. New York; 2019 [citado 05 fev 2018]. Disponível em: https://data.unicef.org/topic/nutrition/low-birthweight/

b Programa das Nações Unidas para o Desenvolvimento; Instituto de Pesquisa Econômica Aplicada; Fundação João Pinheiro. Consulta. Brasília, DF: PNUD; IPEA; Fundação João Pinheiro; 2013 [citado 01 mar 2018]. Disponível em: http://www.atlasbrasil.org.br/2013/pt/consulta/
